# Anti-tobacco control industry strategies in Turkey

**DOI:** 10.1186/s12889-018-5071-z

**Published:** 2018-02-26

**Authors:** Seda Keklik, Derya Gultekin-Karakas

**Affiliations:** 1 Department of Economics, Faculty of Economics and Business, Isik University, 34980, Sile, Istanbul, Turkey; 2 Department of Management Engineering, Faculty of Management, Istanbul Technical University, 34367, Macka, Istanbul, Turkey

**Keywords:** Tobacco, Tobacco industry, Tobacco control, Supply-side tobacco control, Turkey

## Abstract

**Background:**

Transnational tobacco companies (TTCs) penetrated the Turkish cigarette market due to trade and investment liberalization in the post-1980 period and eventually secured full control. Despite tobacco control policies put in place in reaction to accelerating consumption, TTCs reinforced their market power through a variety of strategies. This paper explores industry strategies that counteract tobacco control policies in Turkey.

**Methods:**

The study employs both qualitative and quantitative analyses to explore industry strategies in Turkey. Besides the content analyses of industry and market reports, descriptive analyses were conducted for the sub-periods of 1999–2015. The analyses focus on the market strategies of product innovation, advertisement-promotion, cost management and pricing.

**Results:**

Rising sales of low tar, ultra-low tar, slim, super-slim and flavoured cigarettes indicate that product innovation served to sustain consumption. Besides, the tobacco industry, using its strong distribution channels, the Internet, and CSR projects, were found to have promoted smoking indirectly. The industry also rationalized manufacturing facilities and reduced the cost of tobacco, making Turkey a cigarette-manufacturing base. Tobacco manufacturers, moreover, offered cigarettes in different price segments and adjusted net prices both up and down according to price categories and market conditions. In response to the successful effect of shifts in price margins, the market share of mid-priced cigarettes expanded while those within the economy category maintained the highest market share. As a result of pricing strategies, net sales revenues increased. Aside from official cigarette sales, the upward trends in the registered and unregistered sales of cigarette substitutes indicate that the demand-side tobacco control efforts remain inadequate.

**Conclusions:**

The Turkish case reveals that the resilience of the tobacco industry vis-à-vis mainstream tobacco control efforts necessitates a new policy perspective. Rising market concentration by TTCs and the global nature of industry strategies require that the highly profitable manufacturing and trade of tobacco products should be discouraged on a basis of international collaboration. To reduce and eventually eradicate tobacco consumption, supply-side tobacco control measures are needed along with demand-side policies.

**Electronic supplementary material:**

The online version of this article (10.1186/s12889-018-5071-z) contains supplementary material, which is available to authorized users.

## Background

Liberalization of the tobacco trade and investment under the auspices of the International Monetary Fund (IMF), World Bank (WB) and World Trade Organization (WTO) [1] has made the tobacco epidemic a global phenomenon. As transnational tobacco companies (TTCs) rushed into low- and middle-income countries in the 1980s and 1990s, they acquired privatized public monopolies [2]; followed aggressive marketing strategies; used efficient distribution networks; and extensively increased the number of retail sales points [3]. In response to the rise in tobacco consumption^1^ [1, 4] and tobacco-related deaths [5], the Framework Convention on Tobacco Control (FCTC) [6] was endorsed in 2003 under the auspices of the World Health Organization (WHO). The FCTC and the following policy package “MPOWER”^2^ in 2008 [7] intend to modify the behaviour of (potential) smokers through measures such as sales taxes and advertising bans. On the other hand, smuggling controls, banning sales to minors and alternative crop policies remain to be a few supply-side measures mandated by the FCTC that target the manufacturing and trade of tobacco [4]. Meanwhile, to maintain its existence, the tobacco industry continued to use market strategies of product innovation^3^; advertising^4^; foreign investment^5^; pricing,^6^ and illicit trade^7^ as well as non-market strategies of lobbying^8^ and deceptive and/or manipulative activity in scientific research.^9^ The continuing growth of TTCs [8] has triggered a debate on the effectiveness of demand-side policies and the need for a greater emphasis on supply-side measures in the FCTC.^10^

The penetration of TTCs via exportation and then direct investment in the post-1980 period comprehensively restructured the Turkish tobacco sector [9]. By 2015, five transnational companies −PhilSA, Japan Tobacco International (JTI), BAT, Imperial and European− had acquired 99.5% of the previously state-controlled Turkish cigarette market [10]. Concomitantly, cigarette consumption increased dramatically (Additional file 1), necessitating the adoption of tobacco control measures. The first tobacco control law (No. 4207) was passed in 1996. Despite the lack of a clear penalty clause, the law was successful in terms of restricting smoking in specific places and securing advertising bans and written warnings on packages [11]. However, the ban on sales to minors under the age of 18 was not successfully implemented due to insufficient inspections [12]. Turkey signed the FCTC in 2004, and, having abided by its requirements, a new law (No. 5727) broadening the scope of the previous law was passed in 2008 [12]. This gradually brought a 100% smoke-free regulation to all closed and common places including catering businesses and cabs; it also extended the advertising bans to include tobacco sponsorships. With this step, Turkey became one of the first six countries in the world (UK, Ireland, New Zealand, Uruguay, Bermuda, and Turkey) to have a powerful anti-tobacco law [13]. Tobacco excise taxes were also substantially increased after 2010. These tobacco control policies, however, have had a very limited effect on cigarette consumption which remains far above the level of the early1980s before the tobacco market was opened to TTCs.^11^

It is critical to discuss the Turkish case for several reasons: Turkey’s tobacco market saw a dramatic restructuring with the entry of TTCs: the state monopoly and farming subsidies were abolished; tobacco production decreased while import increased, and consumption accelerated [14]. In the process, Turkey’s anti-tobacco strategies have been rated a success by government authorities [13] and WHO [15]. Despite tobacco control interventions, manufacturing and the trade of tobacco products, however, continue to be a profitable business. Accordingly, there is a need to address the strategies that serve to sustain the existence and profitability of the tobacco industry. On this basis, it will be possible to design policies that could end this sector as a source of profit at the expense of public health. The literature on the Turkish tobacco market is very limited, mostly adhering to chronological accounts which fail to present any in-depth empirical findings and analytical research. To our best knowledge, this is the first article based on a comprehensive data analysis to address various industry strategies in the Turkish cigarette market. Besides, the analysis is enriched with relevant data and information on the registered and unregistered markets of other tobacco products. The paper contributes also to the international literature by addressing a middle-income country case. The elaboration of tobacco industry strategies in Turkey would allow comparisons with the cases of other countries to draw lessons applicable internationally.

## Methods

This study examines the market strategies of product innovation, advertisement-promotion, cost management and pricing for Turkey by employing both qualitative and quantitative analyses. Besides the content analyses of industry and market reports, descriptive analyses were conducted for different periods within the span of 1999–2015 as the data available for specific industry strategies rarely covered the same period. We disregarded non-market strategies because of the difficulty in addressing all industry strategies. Further research can include the fieldwork necessary to fully understand the role of non-market strategies. The analysis focuses on cigarettes − the most commonly used tobacco product in Turkey −, while also shedding light on other tobacco products to evaluate tobacco control efforts. Because of the limited scope of the paper, illicit trade was included merely to show that consumption of tobacco products is higher in Turkey than official cigarette sales indicate.

### Product innovation strategy

We examined the trends in cigarette sales by tar level, thickness and flavour to shed light on the use of product innovation strategy to sustain smoking. The data source was Passport Database of Euromonitor International. Firstly, we compared the sales of high tar, low tar, ultra low tar and mid-tar^12^ cigarettes for the period of 2007–2015. Then, we evaluated the sales of slim and super-slim cigarettes in comparison to regular cigarettes between 2008 and 2015. Thirdly, we explored the changes in sales of cigarettes with flavour in comparison to standard cigarettes between 1999 and 2015.

### Advertisement-promotion strategy

We evaluated advertising-promotion strategy on the basis of the review of the academic literature, as well as company and market reports (Euromonitor). Given the advertising bans, we focused on the use of the indirect channels for the promotion of consumption which are distribution networks, the Internet and CSR projects.

### Cost-management strategy

We firstly reviewed the findings in the academic literature and company reports (Euromonitor) regarding cost management strategy. Then, we focused on changes in the cost of tobacco in the 2000s. Firstly, we calculated decreases in the cost of imported tobacco between 2010 and 2015. To this end, the following data was retrieved from Tobacco and Alcohol Market Regulatory Authority (TAPDK): dollar values of the Tobacco Fund, an excise tax levied on imported tobacco per ton; and, volumes of domestic and imported tobacco used by tobacco manufacturers. We subtracted the Tobacco Fund in the relevant year from the value of the Fund that was imposed in 2010. The difference was multiplied by the quantity of imported tobacco in the relevant year. Afterwards, we calculated the savings from the costs of tobacco over the period. Secondly, we aimed to see whether the industry increasingly provided tobacco from the low-cost regions in Turkey. Thus, by using the data from TAPDK, we explored the regional trends in volumes of domestic tobacco purchases by the manufacturers for the period 2010–2015.

### Pricing strategy

Pricing strategy was analysed for three brand segments: the economy, mid-priced and premium brands. Because of data constraints, the analysis was restricted to the period 2005–2012 and also to the average annual prices per pack of only seven cigarette brands. The premium segment consists of Parliament and Marlboro, both produced by PhilSA; in the mid-priced segment, there is Winston, produced by JTI; and in the economy segment there are Monte Carlo, produced by JTI, and Tekel 2001, Maltepe and Samsun all produced by BAT. The price data was retrieved from Passport Database of Euromonitor International. To calculate real values, we used the Consumer Price Index (CPI) (2003 based) released by Turkish Statistical Institute (TurkStat). The brands were categorized according to the price classification adopted by Euromonitor International: cigarette brands with a price of 10 TL and above per pack were classified as premium; between 8.1–9.99 TL as mid-priced; and, 8 TL and below as economy [16].

**Fig. 1:**
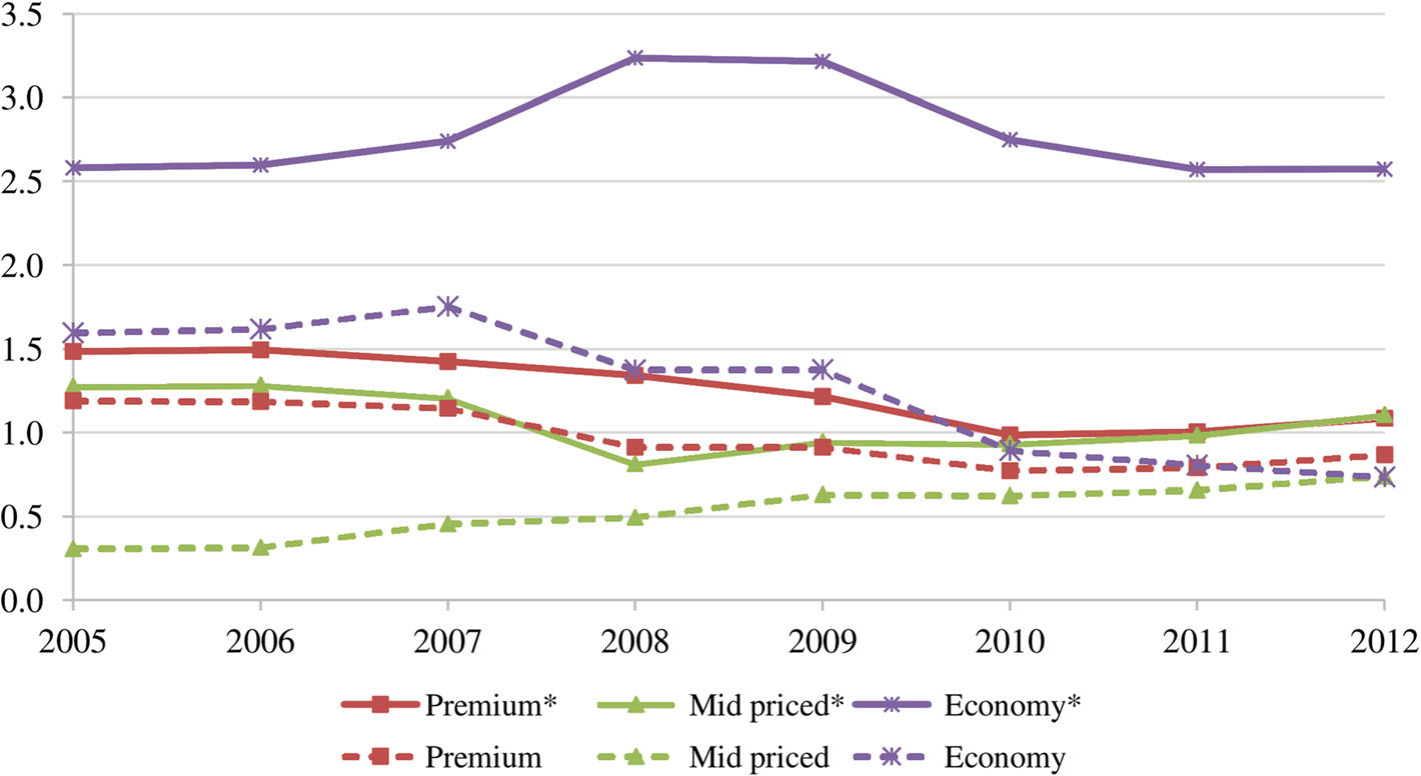
Cigarette sales for the sample and for the total market* by price segment (in billion packs), 2005–2012. Data source: [10]

**Fig. 2:**
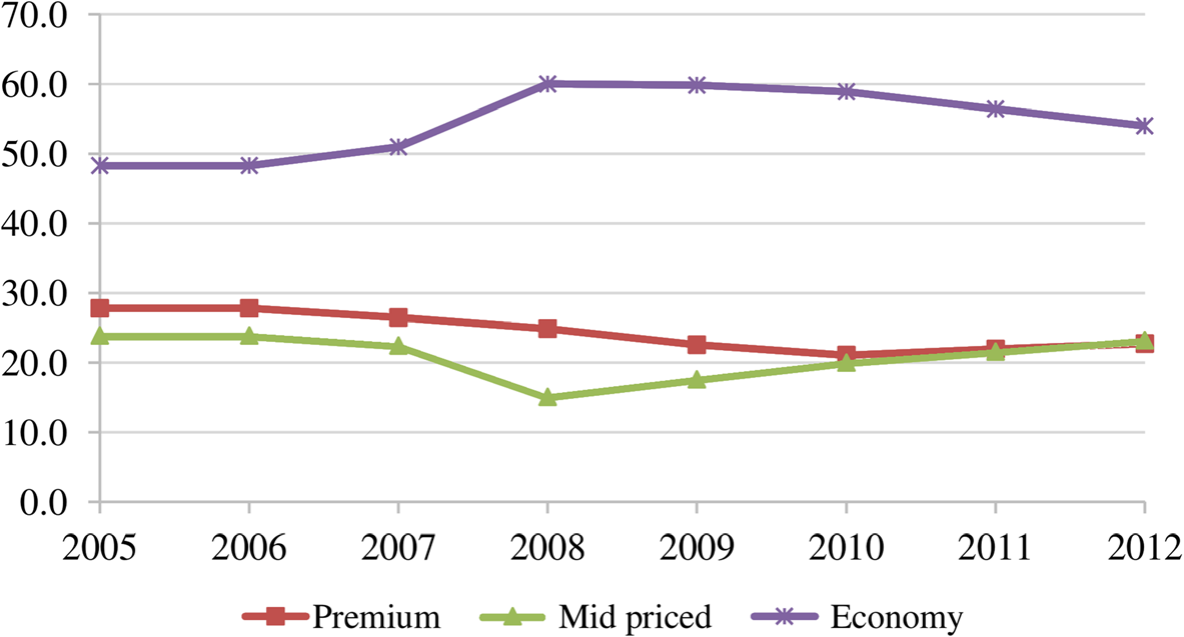
Cigarette market shares by price segment (%), 2005–2012. Data source: [10]

The analysis followed four steps: Firstly, after-tax real weighted prices^13^ for pack of 20 cigarettes were calculated for the three brand segments. Then, to identify the trends in net prices, before-tax (net) real weighted prices^14^ were calculated. Thirdly, we looked at the performance of different price segments in share of market volume. However, because of the limited number of cigarette brands for which the data was available, the sample underrepresented the total cigarette sales in the period under consideration. Indeed, the sample represented the premium cigarette market well; and the representative power of the sample increased for the mid-priced segment after 2006. The market for economy brands, however, became underrepresented by the sample after 2007 (Table 1) since sales volumes of newly introduced economy brands increased (Fig. 1). This situation would have resulted in misrepresentation as it may have been thought that sales volumes and net revenues in the economy segment declined over the years. In fact, the total market data showed that the economy segment achieved the highest sales volume (almost 54% of the market in 2012) (Fig. 2). Therefore, the total market sales data was more reliable than the sample data. Consequently, the changes in before-tax (net) nominal sales revenues for the three brand segments have been examined by taking into account total market sales (Additional file 2). To find net revenues, net nominal weighted prices of the sample brands were multiplied by the total market sales volumes. In the evaluation of the changes in total volume of market sales and net nominal sales revenues, trends in price margins between different brand segments were taken into accounts.

**Table 1:** Share of the sample in total cigarette sales (%), 2005–2012

	Premium	Mid-priced	Economy
2005	80.1	24.1	61.9
2006	79.3	24.5	62.3
2007	80.3	37.8	64.0
2008	68.0	60.9	42.5
2009	75.1	66.6	42.8
2010	78.5	66.9	32.5
2011	78.9	66.9	31.3
2012	79.9	66.7	28.5

*Data source:* [10]

## Results

A limited number of studies present partial findings connected to specific industry strategies in Turkey. The industry interfered via lobbying in the making of tobacco control laws in the 1990s [17, 18]. The neo-liberal transformation of the Turkish tobacco market in favour of TTCs stimulated trade and manufacturing and resulted in accelerated consumption [9, 14]. While some tobacco control policies were put into effect, the industry continued to be supported through tax and investment incentives [19]. Tobacco companies used tax increases to disguise their before-tax nominal price rises, raising their sales revenues in the last years [20, 21]. Violations of bans are pervasive: one out of every four hospitality premises in four districts of Istanbul was observed to be violating the smoke-free law in 2015 [22]; also, rising violations of display rules at points of sale show that the industry uses points of sale as avenues for advertising and promotion targeting the youth and other groups [23]. The comprehensive findings of this research on the Turkish case further support the claim that TTCs continue to manufacture as long as they make a profit, selling more products and in more varieties [4, 24]. Tobacco control policies, therefore, will remain inadequate as long as they fail to target the industry as the supplier of tobacco products [25].

### Product innovation strategy

**Fig. 3:**
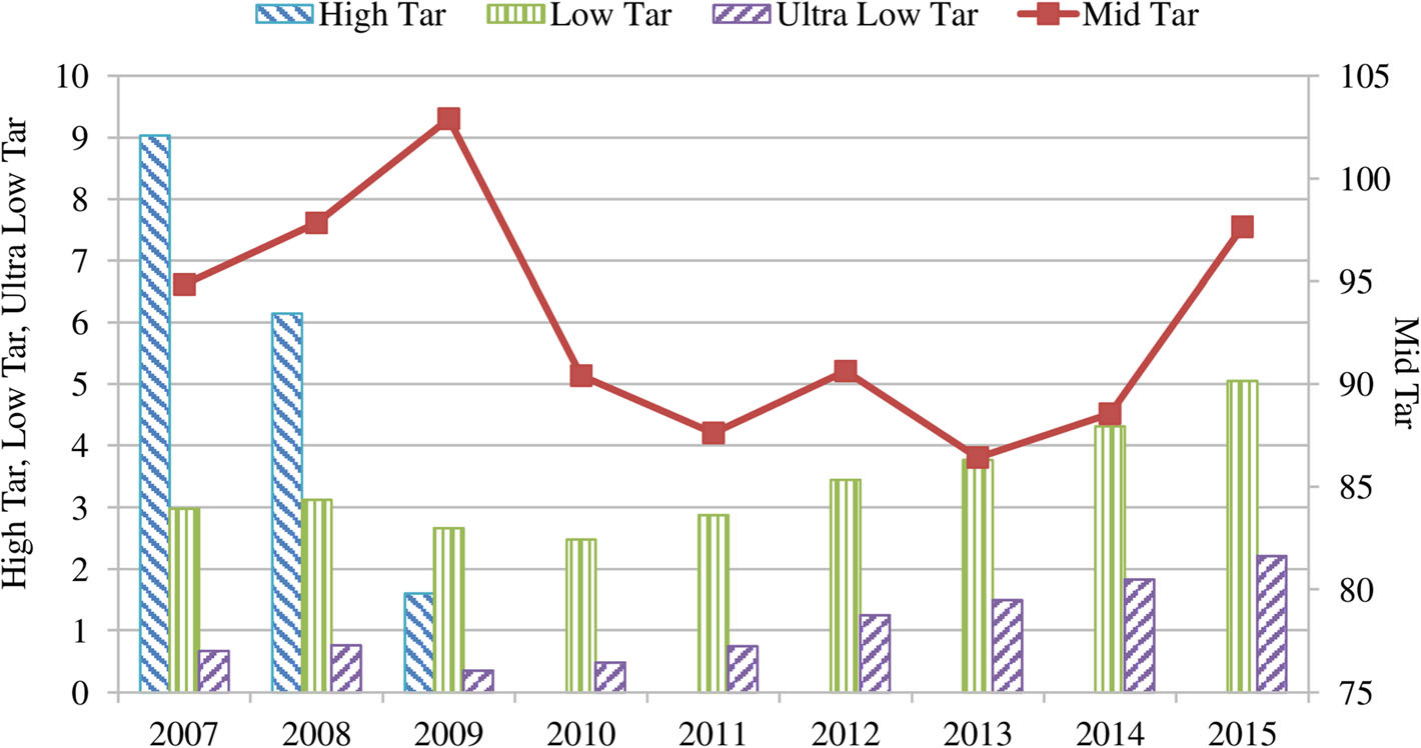
Cigarette sales in Turkey by tar level (billion sticks), 2007–2015. Data source: [10]

TTCs perpetuated smoking firstly by lowering tar levels. Consumers began to prefer reduced tar cigarettes since they perceived them to be less harmful than high-tar cigarettes: The sales of high-tar cigarettes ended by 2010; during the period 2007–2015, sales of mid-tar cigarettes increased by only 2.9% while the rate reached 66.3% for low-tar cigarettes and 204% for ultra-low tar cigarettes (Fig. 3).

**Fig. 4:**
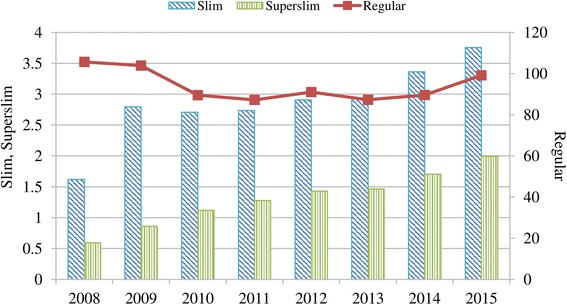
Cigarette sales in Turkey by thickness (billion sticks), 2008–2015. Data source: [10]

Secondly, TTCs released slim and super-slim cigarettes that are longer and contain less tobacco. The sales of regular cigarettes decreased by 6.2% while the sales of slim and super-slim cigarettes increased by 132.1% and 236% respectively during the period 2008–2015 (Fig. 4).

**Fig. 5:**
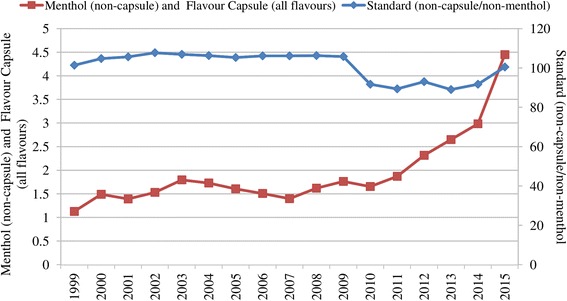
Flavoured and standard cigarette sales in Turkey (billion sticks), 1999–2015. Data source: [10]

Thirdly, cigarettes were made more attractive by adding flavour. As the sales of standard cigarettes had a tendency to decrease in the period 1999–2007, sales of cigarettes with flavour (menthol (non capsule) and flavour capsule (all flavours)) fluctuated until 2007 and then increased by 218% between 2007 and 2015 (Fig. 5).

As a result, rapid product differentiation makes Turkey a special case. While the market for tobacco products had included only a few brands during the state-monopoly period, there were 589 domestically produced and licensed products as of April 2014 [26]. Furthermore, due to insufficient regulation, cigarette manufacturers could use more than 600 additives and consequently designed more attractive and addictive products [26].

### Advertising, promotion and sponsorship strategies

Although FCTC Article 13 [6] forbids all direct and indirect advertising, promotion and sponsorship activities, the Turkish tobacco industry violates this rule. The retail ban on minors under the age of 18 is commonly violated [27] at points of sale. There are about 158 thousand points of sale registered by the TAPDK. Tobacco manufacturers use their own distribution channels to deliver cigarettes to points of sale which include groceries, markets, supermarkets, hypermarkets, kiosks, sellers of dried nuts and specialty outlets that sell only tobacco and alcoholic beverages [9]. The distribution channels play an important part in the operation of the tobacco industry: PhilSA reaches 143 thousand points of sale with 828 employees out of 1613 [28]; JTI reaches 150 thousand points of sale with 132 employees out of 600 [29]; and BAT distributes to 125 thousand points of sale [30]. Employees who work in the sales, marketing and distribution departments of the TTCs in question carry out regular visits to pre-established points of sale, renewing display racks although advertising of tobacco products is forbidden in Turkey [31]. Points of sale are often grouped into four or five segments according to the income level and social status of the neighbourhoods; and, while renewing display racks, different branding and marketing strategies are employed in those segments to increase sales [31].

A recent survey in seven city centres shows that nearly 91.4% of retailers were engaged in at least one type of violation of the display ban [23]. Tobacco product displays at points of sale could not be prevented because of insufficient inspection mechanisms to monitor the wide distribution channels of TTCs [23]. Besides, brand promotion and advertisements were made via the internet and electronic mail; new advertising strategies were developed on social media; and an image of reputable and legitimate corporations was created via CSR projects against prohibitions for direct advertising [9]. For instance, in 2011 PMI allocated 25.3% of its worldwide CSR spending to projects in Turkey [32]. Smoke-free violations in indoor places are also high [9]. The industry tried to build cooperation with the representatives of cafes, hotels and restaurants in order to reverse the indoor smoking ban in 2008 [33].

### Cost management strategy

While TTCs preferred to shut down manufacturing plants in certain countries [34], they selected Turkey as a manufacturing base and rationalized their plants for efficiency. After the purchase of the state monopoly TEKEL in 2008, BAT rationalized the widespread manufacturing network of TEKEL: it closed down most of TEKEL’s plants; and around 12 thousand workers were fired. BAT had located cigarette factories in both Izmir and Samsun, then closed down the Izmir factory (established in 2002) and moved the means of production to the Samsun facility [30]. Likewise, PMI and JTI established high capacity manufacturing plants equipped with the latest technology. The leading manufacturer PhilSA has a factory in Izmir that is the fourth biggest PMI factory in the world with the capacity to manufacture 40 billion sticks per year [28]. Moreover, JTI‘s single factory (established in 1993 in Izmir) can produce 8 to 10 thousand cigarettes per minute, and its annual production is around 35 billion sticks [29]. Also, without any curtailment, the tobacco industry in Turkey can benefit from state subsidies for the production, export and import of tobacco products, helping to reduce costs [19].

TTCs were also able to reduce the cost of tobacco. What facilitated this was firstly the Tobacco Law (No. 4733) passed in 2002 upon the commitment to the IMF. By Law, the state’s subsidized purchases of tobacco had ended and contracted farming started, leaving tobacco farmers without any pricing power to produce and sell at the request of TTCs. The abolition of subsidies and the low pricing policy has driven tobacco farmers into poverty, resulting in a substantial contraction in cultivation [9]. From 1989 to 2015, the population of tobacco farmers and volume of production contracted by 89% and 72% respectively, making Turkey a net importer of tobacco since 2012 [35, 36]. Secondly, a legal amendment to the Tobacco Fund helped the industry to reduce costs. The Fund was levied to improve tobacco cultivation and support farmers in 1986 [37]. In 2008 Turkey made a commitment to eliminate the Fund between 2010 and 2018 as the European Union (EU) argued that it discriminated against imports [9]. The abolition of the Fund allowed TTCs to import American tobacco more cheaply, contributing to industry profitability.

The Fund was reduced from 3000$/tons in 2010, 2250$/tons in 2011, 1800$/tons in 2012, 1500$/tons in 2013, 1200$/tons in 2014, and to 900$/tons in 2015 [38]. As a result, the use of imported tobacco exempted from the Tobacco Fund increased by 61.2% and those purchased with a lowered fund increased by 22.8% in cigarette manufacturing [36].^15^ As TTCs increasingly used imported tobacco, the cost of tobacco decreased significantly during the period 2010–2015 (Table 2). In 2015, the total savings achieved due to the removal or reduction of the Tobacco Fund reached $226,926,300 (=101,025,000 + 125,901,300).

**Table 2:** Cost savings in cigarette manufacturing due to the removal/reduction of the Tobacco Fund, 2010–2015

	Imported Tobacco with removed Tobacco Fund		Imported Tobacco with reduced Tobacco Fund	
	Quantity used (tons)	Decrease in the cost of imported tobacco for cigarette manufacturers ($) (compared to 2010)	Quantity used (tons)	Decrease in the cost of imported tobacco for cigarette manufacturers ($) (compared to 2010)
2010	20,895	62,685,117	–	–
2011	22,536	67,606,706	44,664	33,497,973
2012	29,002	87,006,545	53,981	64,776,785
2013	28,779	86,338,262	52,919	79,378,626
2014	31,573	94,718,086	56,834	102,301,076
2015	33,675	101,025,000	59,953	125,901,300

*Data source:* [92]

**Fig. 6:**
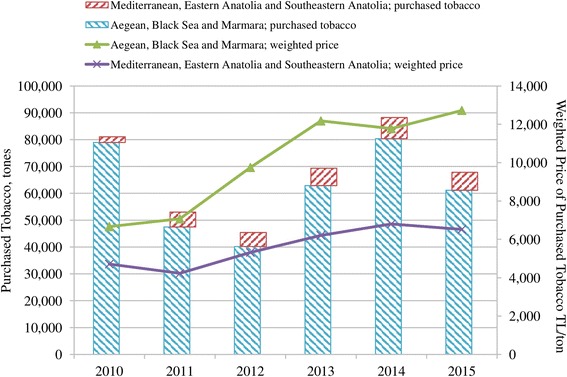
Tobacco purchases by tobacco manufacturers by regions in Turkey, 2010–2015. Data source: [36]

Aside from cost saving through the Tobacco Fund, TTCs were also able to reduce cost by purchasing tobacco at the lowest price. In that sense, the purchases of tobacco from the low-cost Mediterranean, Eastern Anatolia and South-eastern Anatolia Regions increased compared to the lower share of Aegean, Black Sea and Marmara Regions in total domestic tobacco use (Fig. 6).

### Industry pricing strategy

**Fig. 7:**
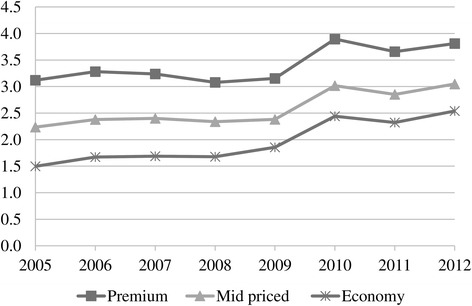
After-tax real weighted prices per pack of cigarettes by price segment, 2005–2012. Data source: [10 93]

The analysis shows the trends in cigarette prices, volumes and revenues by brand segment in Turkey between 2005 and 2012. The after-tax real prices went up in all price segments except in 2008 and 2011 when the global financial and Euro debt crises adversely influenced Turkey (Fig. 7).

**Fig. 8:**
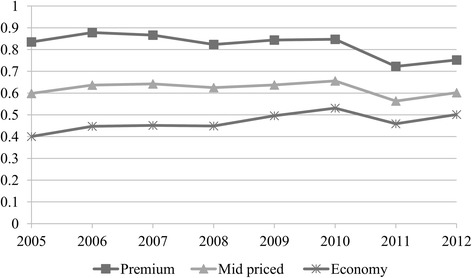
Before-tax (net) real weighted prices per pack of cigarettes by price segment, (TL) 2005–2012

**Fig. 9:**
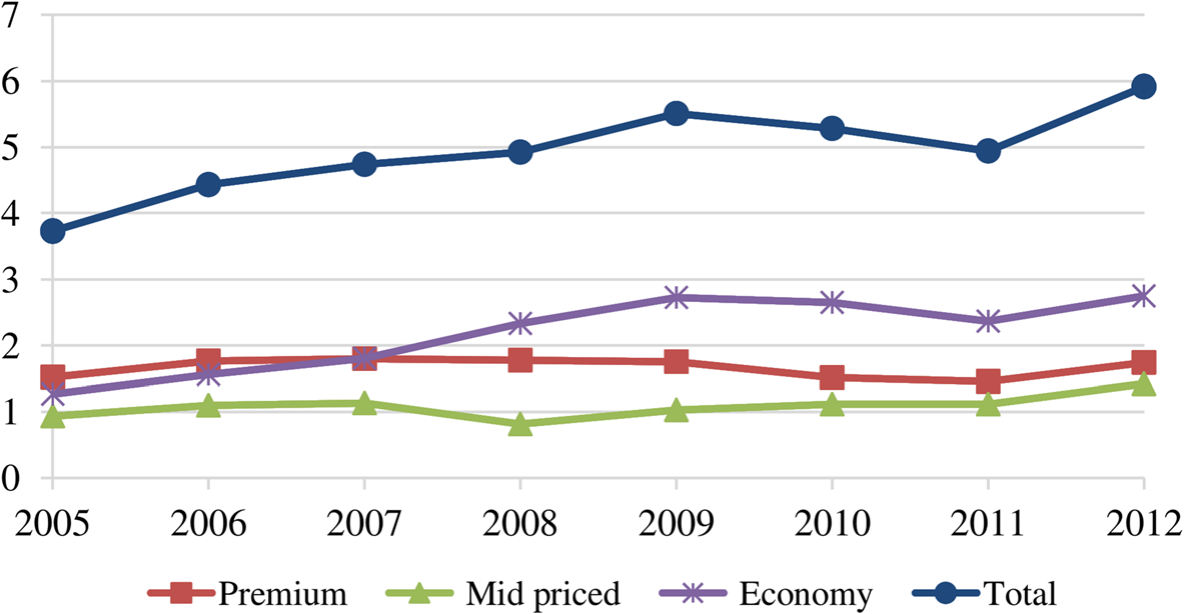
Before-tax (net) nominal sales revenues (billion TL), 2005–2012

Figure 8 depicts before-tax (net) real weighted prices. During 2005–2009 when the minimum specific SCT per pack changed only marginally (Table 3), net real prices tended to rise in all categories, supporting profitability (except for the year 2007 for premium brands and for the crisis year 2008) (Table 4). In 2010 when SCT per pack (% of retail price) increased, the tobacco industry passed on taxes to consumers by increasing net real prices in all price segments. Yet in 2011 when SCT per pack (% of retail price) continued to increase, TTCs lowered net real prices to reduce the amount of tax that consumers had to pay. This increased sales volumes of the mid-priced and premium brands (Fig. 1), limiting the decrease in total sales revenues (Fig. 9). However, in 2012 when taxes remained constant, tobacco manufacturers increased net real prices in all segments (Fig. 8) and due to the higher sales of mid-priced and premium brands, their revenues improved (Fig. 9). As a result, according to market conditions, TTCs adjusted their prices by brand segments to maintain their revenues.

**Table 3:** Sales taxes on cigarettes, 2005–2012

	2005	2006	2007	2008	2009	January 2010	September 2011	November 2011	May 2012
Minimum specific SCT per stick (TL)	0.0675	0.0700	0.0750	0.0775	0.1025	0.1325	–	–	–
Minimum specific SCT per pack (TL) (20 sticks* Minimum specific SCT per stick)	1.35	1.40	1.50	1.55	2.05	2.65	0.00	0.00	–
Specific SCT per pack (TL)	–	–	–	–	–	–	–	–	–
SCT per pack (% of retail price)	58.00%	58.00%	58.00%	58.00%	58.00%	63.00%	69.00%	65.00%	65.00%
VAT per pack (% of retail price)	18.0%	18.0%	18.0%	18.0%	18.0%	18.0%	18.0%	18.0%	18.0%

*Data source:* [38]

**Table 4:** Change in before-tax (net) real weighted prices per pack of cigarettes by price segment (TL), 2005–2012

	Premium	Mid-priced	Economy
2005–2006	0.043	0.038	0.047
2006–2007	−0.012	0.005	0.004
2007–2008	−0.043	−0.017	−0.003
2008–2009	0.020	0.012	0.047
2009–2010	0.004	0.019	0.035
2010–2011	−0.125	−0.093	−0.072
2011–2012	0.030	0.039	0.043

**Fig. 10:**
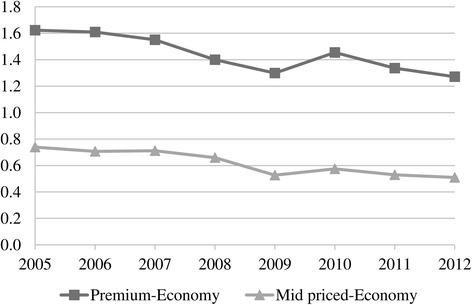
Price margins between premium and economy brands and between mid-priced and economy brands (TL), 2005–2012. * Calculated with real weighted prices

While TTCs were adjusting prices to offset rises in tax, the tobacco industry gathered the highest net nominal sales revenue in the economy segment during 2007–2012 (Fig. 9). This is because economy brands had the highest market share (Fig. 2) despite their unit revenue being low. Even though they had a similar market share to mid-priced brands (Fig. 2), premium brands accrued more revenues (Fig. 9) due to higher per unit revenue. Furthermore, sales revenues from the mid-priced segment increased after 2008 (Fig. 9) accompanying the rise in its market share (Fig. 2). This outcome was related to the changes in price margins. The price margins between the premium and economy brands as well as between the mid-priced and economy brands tended to decrease especially after 2007 (Fig. 10). Along with the declining price margins, consumers tended to shift to mid-priced brands, leading to the rising market share in this segment (Fig. 2). Meanwhile, the market share of the economy brands decreased after 2009 while the fall in the share of premium brands stopped in 2011 (Fig. 2). Lastly, in spite of the adoption of tobacco control policies, the trend of increasing total sales revenues was noteworthy (Fig. 9).

Even though our analysis had to be restricted to the period of 2005–2012, some points about pricing strategy pursued after 2012 can be emphasized. Firstly, tobacco manufacturers took advantage of the existing tax structure in Turkey. In the process of harmonization with EU regulations, a specific tax was added per cigarette pack as of 1 January 2013 and this tax increased over the years (Table 5). However, ad valorem taxes (SCT and VAT) account for more than 80% of the 10 TL retail price of a packet of cigarettes while specific tax accounts for a very small ratio of only 2% of the total tax burden. Bakir indicates that the very high ad valorem taxes in contrast to the EU led tobacco manufacturers in Turkey to price competition [39]. This increased after mid-2013, especially for some mid-priced and premium brands. As a result, consumption was encouraged due to the lowered tax burden on consumers. Moreover, to avoid tax increases, price-sensitive consumers tended to shift to cheaper brands because of their lower ad valorem taxes. Due to the increases in the specific tax per pack (Table 5), moreover, low-priced cigarettes became more expensive and thus demand for the mid-priced brands was boosted [39].

**Table 5:** Sales taxes on cigarettes, 2012–2016

	January 2013	July 2013	January 2014	July 2014	January 2015	July 2015	January 2016
Minimum specific SCT per stick (TL)	0.1575	0.1613	0.1875	0.1971	0.1994	0.2103	0.2210
Minimum specific SCT per pack (TL) (20 sticks* Minimum specific SCT per stick)	3.15	3.23	3.75	3.94	3.99	4.21	4.42
Specific SCT per pack (TL)	0.0900	0.0922	0.1300	0.1366	0.1866	0.1968	0.2468
SCT per pack (% of retail price)	65.25%	65.25%	65.25%	65.25%	65.25%	65.25%	65.25%
VAT per pack (% of retail price)	18.0%	18.0%	18.0%	18.0%	18.0%	18.0%	18.0%

*Data source:* [38]

### Consumption of tobacco products in Turkey

Although total registered cigarette sales did at first decrease marginally between 2008 and 2012 in response to tobacco control interventions, as a result of the above-discussed industry activities, they persistently increased from 91.6 to 105.5 billion sticks between 2013 and 2016. SCT revenues from tobacco products meanwhile consistently increased from 20 to 25 billion Turkish Lira between 2003 and 2015 and the share of tobacco products in total SCT revenues reached 26% in 2015 [40].

**Fig. 11:**
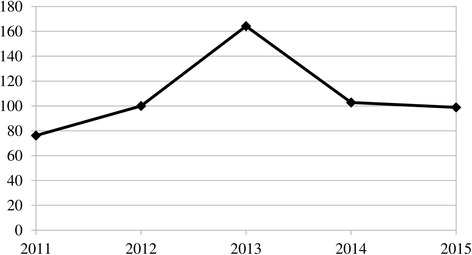
RYO tobacco sales in Turkey (tons = million sticks), 2011–2015. Data source: [10]

In the discussion of the effectiveness of tobacco control policies, there is a need to consider the consumption of contraband products. It is estimated that 10–15% of cigarette consumption in Turkey was based on illegal products during the period 2002–2007 [33]. This shows that actual consumption has been much higher than the official cigarette sales indicate.^16^ Moreover, as a result of increasing sales taxes,^17^ price-responsive consumers tend to use tobacco that consists of cut tobacco sold in a packaged format for use in roll-your-own (RYO) cigarettes (Fig. 11). RYO tobacco is preferred to cigarettes especially among young smokers as it is cheaper [41]. Figure 11 only depicts official sales, but industry experts believe that the total size of the RYO tobacco market - including illicit sales - was around 14,000 tons in 2014, although no official data for illicit trade exists [41]. Therefore, for 2014, total cigarette consumption increased from 94.7 to around 122.7 billion sticks when the sales of official and illicit RYO tobacco (1 g = 1 cigarette stick) as well as contraband cigarettes^18^ are considered.

Next, there is the need to regard the consumption of non-cigarette tobacco products that are currently manufactured by domestic firms in Turkey. Firstly, waterpipe smoking (hookah) has accelerated worldwide since the 1990s but most markedly in the Middle East and North Africa. Waterpipe tobacco (WT) is falsely marketed as being less harmful than cigarettes. In reality, it makes it more difficult to quit cigarette smoking and leads young adolescents to start cigarette consumption [42]. Drawing on the Global Adult Tobacco Survey (GATS), it is claimed that overall waterpipe smoking prevalence declined in Turkey between 2008 and 2012 from 2.3% to 0.8% [43]. However, the prevalence in the 15–24 age group was much higher (4.3%) and the prevalence did not decline among females and non-graduates as the GATS found in 2012 [44]. Waterpipe smoking is spreading especially among the younger population; GATS shows that the use of cigarettes and other tobacco products among the young in Turkey increased by 51% and 88% respectively between 2003 and 2012 [44]. New studies raise serious concerns about the rapidly growing hookah sector in the country: illicit products constitute 99% of the total WT consumption, revealing that the informal economy dominates the WT market [42, 45]. It is estimated that 15,500 waterpipe smoking businesses were in operation while the number of TAPDK registered businesses was only 1220 as of October 2016 [42]. Also, while the official annual production volume of WT was only 9 tons in Turkey, the total annual consumption was estimated approximately 1500 tons in 2015 [42]. Rising consumption, therefore, was based on both domestically produced and imported contraband WT [42, 45]. All these point to significant inspection and enforcement failures in the Turkish WT market.

**Fig. 12:**
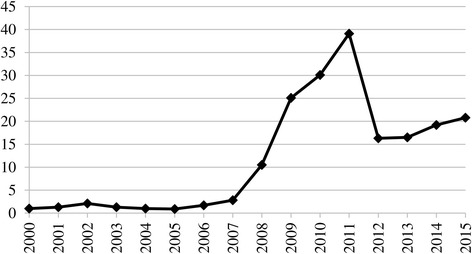
Cigar and cigarillo sales in Turkey (million units), 2000–2015. Data source: [10]

The consumption of cigars and cigarillos has also been a rising trend in Turkey. As a consequence of the TAPDK’s lifting of the restriction on the import of cigars and cigarillos by non-manufacturing companies in 2011, the import of foreign brands and consumption was found to have increased dramatically by 2012 (Fig. 12). In that year TAPDK stipulated that only those cigars and cigarillos with specific ingredients conforming to the standards released by the Turkish Standards Institute could be sold [46]. As a result in 2012 official sales decreased while the sales of the products that could not be imported increased in the contraband cigar and cigarillo market [47]. As reported by trade experts, around 85% of the sales of cigars and cigarillos in 2013 were illegal [47]. Therefore, the decrease in official sales of cigar and cigarillo after 2011 masks the high magnitude of real consumption (Fig. 12).

## Discussion

Tobacco control policies implemented to confront the tobacco epidemic directly conflict with tobacco companies’ raison d’être [25]. The industry always tries to counteract tobacco control policies implicitly [14]. Various countries’ experiences show that the industry either acts to prevent the adoption of tobacco control policies, or if it cannot prevent them, aims to undermine them [34, 48–53]. The literature reveals that in the face of tobacco control measures, tobacco manufacturers maintain their existence by pursuing a variety of tactics.

The overall results are compatible with global tobacco industry strategies addressed in the literature. The pursuit of multiple strategies is also valid in the Turkish case, making tobacco control policies largely ineffective. Government interventions in the form of sales taxes and advertising bans etc. are nullified by a tobacco industry that continues to innovate products, finds indirect ways to advertise, manipulates excise tax increases for higher sales revenues as well as manages costs in multiple ways. Turkey should therefore utilize supply-side measures that directly target the trade and manufacturing activities of tobacco companies.

The industry in Turkey also perpetuates smoking addiction via product innovation. What distinguishes Turkey, however, is rapid product innovation. Since there is no standardization of cigarette packages and sticks, it creates more room for product innovation [14]. Product innovation and use of additives in tobacco products, therefore, should be banned and plain packaging needs to be adopted.

The use of indirect tactics for advertising, promotion and sponsorship in Turkey is also similar to cases in other countries with restrictive tobacco control measures, but the bans on sales to minors, product display and indoor smoking have been widely circumvented in Turkey. The very high number of points of sale in Turkey has to be reduced and sales of tobacco products must be restricted to only specialty outlets. The sales ban to minors under the age of 18 should come into full force.

In regard to cost management strategies there are further features specific to Turkey: The removal of the Tobacco Fund on imported tobacco, use of government investment subsidies and the privatization of the state monopoly TEKEL have helped TTCs secure greater profitability. All kinds of subsidies to the tobacco industry should consequently end and negative incentives should be imposed on the industry such as higher corporate tax rates.

Similar to many other countries, Turkey has a high market concentration in cigarette manufacturing which ensures pricing power and profitability for TTCs. Our detailed analysis of pricing strategy shows that without before-tax price controls, the industry is able to use tax and market structures to manipulate consumers, nullifying tax interventions by the authorities. Therefore, to increase the effectiveness of tax policies, policy makers need to consider the pricing strategies pursued by tobacco manufacturers. The high share of ad valorem taxes in cigarette prices allows the manufacturers to engage in price competition in the upper price segments, and thereby undermine tobacco control. Therefore, to discourage price competition, sales taxes need to be based mainly on specific taxes rather than ad valorem taxes. Additionally, to compliment sales tax increases, ceilings on before-tax prices of cigarettes can help to prevent hidden price increases by manufacturers. Lastly, consideration should be given to the possibility that tobacco-trading and manufacturing companies could be publically operated.

Moreover, accelerating trends in the registered and unregistered sales of other tobacco products make Turkey a special case. Aside from official cigarette sales, the upward trend in the registered and unregistered sales of cigarette substitutes indicate that tobacco control efforts remain inadequate in terms of seriously diminishing tobacco consumption in the country. The instability in neighbouring countries such as Iraq and Syria has facilitated the ease with which contraband tobacco products are traded. The unregistered trade also has a tendency to increase because of excise tax increases on cigarettes and the fight against the contraband cigarette trade. Control over the tobacco market should therefore be reinforced via strengthened inspection mechanisms and a more efficient fight against contraband trade.

The study had some limitations. Data constraints made it difficult to analyse industry strategies in the case of Turkey at a finer level. In contrast to advanced economies, tobacco manufacturers in Turkey do not have to publicize their financial data, as they are not publicly open companies. Obtaining appropriate price data was the main limitation in analysing industry strategies. Monthly price data for a broader range of cigarette brands from TAPDK and TurkStat was unavailable. The analysis of pricing strategy, therefore, had to be restricted to the average annual prices of only seven cigarette brands for the period 2005–2012. This made it impossible to examine the changes in before-tax prices in the months that sales taxes were increased. It is recommended that TAPDK make brand-specific price data on a monthly basis available to researchers so that tobacco prices can be more closely monitored.

## Conclusion

In the face of the powerful global oligopolies in the world tobacco market, policy suggestions need to be developed on the basis of shared experiences and implemented on an international scale. To this end, this paper has provided findings from the Turkish case. It is essential to insist on the following truth concerning the dominant paradigm in tobacco control: the profit-oriented tobacco industry is the main responsible party for the diseases and deaths caused by its products, in Turkey as in the rest of the world. In this sense fighting against the tobacco epidemic by holding the tobacco-addicted persons as the main responsible actors for reduction in tobacco consumption will be inadequate. To end the epidemic, supply-side policies pointing to the tobacco industry as chiefly responsible also need to be implemented. Such policies are largely excluded from the realm of tobacco control by reason of their lack of efficacy [54]. In reality, supply-side interventions are disregarded as they would eliminate a significant source of profit and tax revenues. In order to improve the efficacy of anti-tobacco strategies the WHO needs to put supply-side policy measures on its agenda, take steps in this direction and include these in the FCTC. Those countries that have already put some supply-side measures on their agendas should be protected from interference from the tobacco industry via the WTO [55]. Tobacco and tobacco products should be excluded from international trade agreements and their trade should be prevented. In the long run, tobacco manufacturing and trading companies should be transformed into non-profit institutions or organizations with the termination of tobacco consumption viewed as an attainable goal.

## Additional files

Additional file 1: Domestic cigarette sales in Turkey (billion sticks), 1970–2015. Data Source: [92] (DOCX 17 kb)

Additional file 2: Retail sales volumes of cigarettes by price segment- total market (billion packs), 2005–2012. Data Source: [10]. (DOCX 17 kb)

Additional file 3: Nominal average prices per pack of cigarettes by price segment (TL), 2005–2012. Data Source: [93] (DOCX 17 kb)

Additional file 4: Retail sales volumes of cigarettes by brand (billion packs), 2005–2012. Data Source: [10]. (DOCX 15 kb)

Additional file 5: Nominal sales revenues of cigarettes by price segment (billion TL), 2005–2012. (DOCX 14 kb)

Additional file 6: Nominal sales revenues of cigarettes by price segment- sample (billion TL), 2005–2012. (DOCX 14 kb)

Additional file 7: Retail sales volumes of cigarettes by price segment- sample (billion packs), 2005–2012. (DOCX 14 kb)

Additional file 8: Nominal weighted prices per pack of cigarettes by price segment (TL), 2005–2012. (DOCX 14 kb)

Additional file 9: Real weighted prices per pack of cigarettes by price segment (TL), 2005–2012. (DOCX 14 kb)

Additional file 10: Before-tax (net) nominal weighted prices per pack of cigarettes by price segment, (TL) 2005–2012. (DOCX 14 kb)

Additional file 11: Before-tax (net) real weighted prices per pack of cigarettes by price segment, (TL) 2005–2012. (DOCX 14 kb)

## Abbreviations

BAT: British American Tobacco
CPI: Consumer Price Index
CSR: Corporate Social Responsibility
EU: European Union
FCTC: Framework Convention on Tobacco Control
GATS: Global Adult Tobacco Survey
IMF: International Monetary Fund
JTI: Japan Tobacco International
KT&G: Korea Tobacco & Ginseng
PhilSA: Philip Morris-Sabanci Holding Partnership
PMI: Philip Morris International
RYO: Roll-your-own
SCT: Special consumption tax
TL: Turkish Liras
TTCs: Transnational tobacco companies
TurkStat: Turkish Statistical Institute
USA: United States of America
VAT: Value added tax
WB: World Bank
WHO: World Health Organization
WT: Waterpipe tobacco
WTO: World Trade Organization

## Footnotes

1 Cigarette consumption increased from 10 billion to 2.2 trillion sticks between 1880 and 1960, and reached 5.7 trillion sticks in 2000 concurrent with the process of trade and investment liberalization [5].

2 MPOWER consists selected demand-side measures of the FCTC: monitor tobacco use and prevention policies; protect people from tobacco smoke; offer help to quit tobacco use; warn about the dangers of tobacco; enforce bans on tobacco advertising, promotion and sponsorship; and raise taxes on tobacco.

3 Through the changes in packaging and with cigarettes [34, 52, 56–61], product innovation maintains an international brand’s image and persuades poorer smokers not to downgrade to cheaper brands, give up smoking or switch to illicit trade [62]. Also, as alternatives to cigarettes, smokeless tobacco (chewing tobacco and snuff) and electronic cigarettes allow users to evade the indoor smoking ban [63–65]; and they can delay quitting smoking [66].

4 The tobacco industry escapes the advertising, promotion and sponsorship ban by developing unconventional new methods [58, 67]. The industry uses brand stretching; brand sharing; product placement; promotion and distribution of sample products; promotions and sales via electronic mail, the Internet and text messages; and sponsoring sport, music, fashion and art activities [24]. CSR campaigns are organized to restore corporate image and get involved in policymaking processes [25, 52].

5 TTCs use foreign investment to gain a share in new markets [52]. The acquisition of privatized local tobacco companies by the TTCs leads to aggressive marketing, effective distribution and lower prices, all of which increase the demand for cigarettes [51, 68–70]. They also sustain profitability via continuous cost management [9, 51, 52].

6 The high market concentration in the global tobacco sector [71] ensures pricing power for a small number of TTCs [72, 73]. The effectiveness of sales taxes in terms of reducing tobacco consumption depends, in part, on pricing strategy [71]. Higher sales taxes generally make tobacco products more expensive, but the level of price increase depends on the decisions by manufacturers regarding how much of the tax will be passed on to consumers [74]. Tobacco manufacturers may increase retail cigarette prices over and above sales tax increases, leading to an overshifting of taxes; or they may decrease retail prices, causing an undershifting of taxes. Limited empirical evidence shows mixed results: tobacco manufacturers have preferred to apply one of the two strategies in various national markets at different times according to the price segments of their brands, industry structure, tax structure etc. [53, 74]. In general, TTCs use low prices in emerging markets to reach as many consumers as they can [24] while they offer products at various price segments in established markets [59] to reach customers with different income levels [34].

7 Illicit trade is an important strategy among TTCs’ market entry tactics [52, 75–77]. It is used to launch their products in nearby economies, to circumvent sales tax increases, and to maintain access of the youth and low-income consumers with cheap tobacco products [52].

8 Lobbying serves to obtain market access, to block or delay implementation of tobacco-control policies and to manipulate individual policymakers and political parties to influence tobacco control policy making agendas [24, 48, 67, 78, 79].

9 The industry also uses deceptive and/or manipulative interference in scientific research: it manipulates the results of studies which prove that tobacco products cause cancer, that second hand smoke is harmful, and that nicotine is an addictive ingredient, by financing studies that claim the opposite [24, 57, 78, 80, 81].

10 The supply-side proposals in the literature have a range of foci: they target product specifications, the marketing-sales end, manufacturing conditions, or both manufacturing and trade of tobacco products. The following are some examples of supply-side policies: reducing nicotine content in cigarettes, controlling tobacco marketing through a public agency, imposing progressive limits on the amount of commercial tobacco released for legal sale, controlling before-tax prices, and transferring responsibility for the supply of cigarettes to a sole supplier and distributor of tobacco products not driven by profit [4, 25, 72, 82–91].

11 For a review of the entries of foreign tobacco companies into the Turkish market and accompanying tobacco control measures see [14].

12 Although tar levels – and what is considered high, medium and low tar - vary across countries, for the sake of consistency when making comparisons, the following definitions are applied to Euromonitor data: high tar cigarettes = tar content of greater than 10 mg; mid tar cigarettes = tar content of 6–10 mg, low tar cigarettes = tar content of 4–6 mg; ultra low tar cigarettes = tar content of less than 4 mg [10].

13 Firstly, nominal prices (Additional file 3) were multiplied by the sales quantities of the cigarette brands (Additional file 4) to find out nominal sales revenues for each brand (Additional file 5). Next, nominal sales revenues and sales quantities of the brands by price segments were aggregated (Additional file 6 and Additional file 7 respectively). Then, nominal weighted prices (Additional file 8) were calculated by dividing nominal sales revenues to sales quantities of cigarette brands for each price segment. Finally, real weighted prices (Additional file 9) were determined using the Turkey’s CPI (2003 = 100) for Turkey.

14 Before-tax (net) nominal weighted prices (Additional file 10) are calculated by deducting the tax amount from nominal weighted prices (Additional file 8) using the formula below. Then, before-tax (net) nominal weighted prices are calculated in real terms by using Turkey’s CPI (2003 = 100) index and net real weighted prices (Additional file 11) are found out. Before-tax (net) nominal weighted price = N - [(N × SCT) + (N × VAT) + (S)] N: Nominal weighted prices S: Amount of specific tax (if any) for relevant year SCT: Special consumption tax VAT: Value added tax

15 The Tobacco Fund was removed from the import of ‘homogenized tobacco or reconstituted tobacco’, ‘expanded tobacco’, ‘expanded stem’ and any kind of tobacco. The Fund was reduced for imported leaf tobacco.

16 Indeed, in the 2010s, the diminished border security because of the ongoing war in Syria and higher consumption prevalence of around 2 million immigrants than the average of Turkey contributed to the increase in both official and contraband trading of tobacco and tobacco products [21]. However, due to recently strengthened border controls, the fight against smuggling of tobacco products has intensified and significant smuggling have been curtailed [45]. This partially shifted consumption from contraband to registered tobacco products. Henceforth, the rise in official cigarette sales between 2013 and 2016 from 91.6 to 105.5 billion sticks was noteworthy. In the rising official sales, the loose implementation of tobacco control measures, insufficient inspection has especially played a role.

17 The ratio of excise taxes in resale prices has risen dramatically: for example, for a cigarette package sold at 8 TL, it was 40.88% in 2009, 48.38% in 2010, 80.25% in 2011, 81.66% in 2013 and 82.84% in 2015 [21].

18 For example, when one adds to the official sales (94.7 billion sticks), the consumption of contraband cigarettes (around 14% of the official sales - 14 billion sticks), brought total cigarette consumption 108.7 billion sticks for 2014.
