# Supplementary material for: Anti-tobacco control industry strategies in Turkey
Source: BMC Public Health. 2018 Feb 26;18:282. doi: 10.1186/s12889-018-5071-z (PMC5828147; doi:10.1186/s12889-018-5071-z)
Supplement: Supplementary file 1 — Domestic cigarette sales in Turkey (billion sticks), 1970–2015. Data Source: [92] (DOCX 17 kb) [file 12889_2018_5071_MOESM1_ESM.docx]

Additional file 1: Domestic cigarette sales in Turkey (billion sticks), 1970-2015.

| **1970** | 39.40 |
| --- | --- |
| **1975** | 52.20 |
| **1980** | 57.00 |
| **1984** | 63.00 |
| **1985** | 63.00 |
| **1990** | 73.30 |
| **1991** | 76.50 |
| **1992** | 78.50 |
| **1993** | 88.40 |
| **1994** | 91.30 |
| **1995** | 95.80 |
| **1996** | 96.60 |
| **1997** | 101.10 |
| **1998** | 108.60 |
| **1999** | 114.40 |
| **2000** | 111.70 |
| **2001** | 111.80 |
| **2002** | 110.00 |
| **2003** | 108.16 |
| **2004** | 108.87 |
| **2005** | 106.72 |
| **2006** | 107.91 |
| **2007** | 107.45 |
| **2008** | 107.86 |
| **2009** | 107.55 |
| **2010** | 93.35 |
| **2011** | 91.22 |
| **2012** | 99.26 |
| **2013** | 91.66 |
| **2014** | 94.68 |
| **2015** | 103.21 |
