# Supplementary material for: Anti-tobacco control industry strategies in Turkey
Source: BMC Public Health. 2018 Feb 26;18:282. doi: 10.1186/s12889-018-5071-z (PMC5828147; doi:10.1186/s12889-018-5071-z)
Supplement: Supplementary file 2 — Retail sales volumes of cigarettes by price segment- total market (billion packs), 2005–2012. Data Source: [10]. (DOCX 17 kb) [file 12889_2018_5071_MOESM2_ESM.docx]

Additional file 2: Retail sales volumes of cigarettes by price segment- total market (billion packs), 2005-2012.

|  | **Premium** | **Mid-priced** | **Economy** |
| --- | --- | --- | --- |
| **2005** | 1.49 | 1.28 | 2.58 |
| **2006** | 1.50 | 1.29 | 2.60 |
| **2007** | 1.42 | 1.21 | 2.74 |
| **2008** | 1.34 | 0.81 | 3.24 |
| **2009** | 1.22 | 0.95 | 3.22 |
| **2010** | 0.98 | 0.93 | 2.75 |
| **2011** | 1.00 | 0.99 | 2.57 |
| **2012** | 1.08 | 1.11 | 2.57 |
