# Supplementary material for: Anti-tobacco control industry strategies in Turkey
Source: BMC Public Health. 2018 Feb 26;18:282. doi: 10.1186/s12889-018-5071-z (PMC5828147; doi:10.1186/s12889-018-5071-z)
Supplement: Supplementary file 3 — Nominal average prices per pack of cigarettes by price segment (TL), 2005–2012. Data Source: [93] (DOCX 17 kb) [file 12889_2018_5071_MOESM3_ESM.docx]

Additional file 3: Nominal average prices per pack of cigarettes by price segment (TL), 2005-2012.

|  | **Brands** | **2005** | **2006** | **2007** | **2008** | **2009** | **2010** | **2011** | **2012** |
| --- | --- | --- | --- | --- | --- | --- | --- | --- | --- |
| **Premium** | Parliament | 3.89 | 4.49 | 4.77 | 5 | 5.4583 | 7.24 | 7.48 | 8.25 |
|  | Marlboro | 3.8 | 4.38 | 4.69 | 4.8958 | 5.3325 | 6.97 | 7.23 | 8 |
| **Mid- Priced** | Winston | 2.74 | 3.2 | 3.5 | 3.75 | 4.0708 | 5.48 | 5.73 | 6.5 |
| **Economy** | Monte Carlo | 2.11 | 2.3 | 2.51 | 2.75 | 3.2083 | 4.49 | 4.64 | 5.42 |
|  | Tekel 2001 | 2.21 | 2.58 | 2.82 | 3.0299 | 3.3733 | 4.48 | 4.73 | 5.5 |
|  | Maltepe | 1.46 | 1.91 | 2.15 | 2.3073 | 2.8537 | 4.19 | 4.39 | 5 |
|  | Samsun | 1.48 | 1.93 | 2.17 | 2.3322 | 2.8661 | 4.19 | 4.39 | 5 |
