# Supplementary material for: Anti-tobacco control industry strategies in Turkey
Source: BMC Public Health. 2018 Feb 26;18:282. doi: 10.1186/s12889-018-5071-z (PMC5828147; doi:10.1186/s12889-018-5071-z)
Supplement: Supplementary file 4 — Retail sales volumes of cigarettes by brand (billion packs), 2005–2012. Data Source: [10]. (DOCX 15 kb) [file 12889_2018_5071_MOESM4_ESM.docx]

Additional file 4: Retail sales volumes of cigarettes by brand (billion packs), 2005-2012.

|  | **Brands** | **2005** | **2006** | **2007** | **2008** | **2009** | **2010** | **2011** | **2012** |
| --- | --- | --- | --- | --- | --- | --- | --- | --- | --- |
| **Premium** | Parliament | 0.38 | 0.37 | 0.47 | 0.38 | 0.42 | 0.33 | 0.37 | 0.43 |
|  | Marlboro | 0.81 | 0.81 | 0.68 | 0.54 | 0.50 | 0.44 | 0.42 | 0.44 |
| **Mid- Priced** | Winston | 0.31 | 0.32 | 0.46 | 0.50 | 0.63 | 0.62 | 0.66 | 0.74 |
| **Economy** | Monte Carlo | 0.03 | 0.04 | 0.04 | 0.04 | 0.23 | 0.23 | 0.21 | 0.24 |
|  | Tekel 2001 | 0.77 | 0.79 | 0.78 | 0.70 | 0.68 | 0.53 | 0.50 | 0.41 |
|  | Maltepe | 0.43 | 0.43 | 0.43 | 0.34 | 0.21 | 0.06 | 0.04 | 0.04 |
|  | Samsun | 0.37 | 0.36 | 0.50 | 0.29 | 0.26 | 0.08 | 0.06 | 0.05 |
