# Supplementary material for: Anti-tobacco control industry strategies in Turkey
Source: BMC Public Health. 2018 Feb 26;18:282. doi: 10.1186/s12889-018-5071-z (PMC5828147; doi:10.1186/s12889-018-5071-z)
Supplement: Supplementary file 5 — Nominal sales revenues of cigarettes by price segment (billion TL), 2005–2012. (DOCX 14 kb) [file 12889_2018_5071_MOESM5_ESM.docx]

Additional file 5: Nominal sales revenues of cigarettes by price segment (billion TL), 2005-2012.

|  | **Brands** | **2005** | **2006** | **2007** | **2008** | **2009** | **2010** | **2011** | **2012** |
| --- | --- | --- | --- | --- | --- | --- | --- | --- | --- |
| **Premium** | Parliament | 1.48 | 1.68 | 2.23 | 1.88 | 2.28 | 2.39 | 2.75 | 3.55 |
|  | Marlboro | 3.07 | 3.56 | 3.17 | 2.63 | 2.64 | 3.08 | 3.06 | 3.49 |
| **Mid- Priced** | Winston | 0.85 | 1.01 | 1.60 | 1.86 | 2.57 | 3.42 | 3.78 | 4.81 |
| **Economy** | Monte Carlo | 0.07 | 0.09 | 0.11 | 0.10 | 0.73 | 1.02 | 0.97 | 1.29 |
|  | Tekel 2001 | 1.69 | 2.03 | 2.19 | 2.12 | 2.28 | 2.36 | 2.36 | 2.25 |
|  | Maltepe | 0.63 | 0.82 | 0.93 | 0.79 | 0.60 | 0.26 | 0.18 | 0.20 |
|  | Samsun | 0.54 | 0.70 | 1.09 | 0.68 | 0.75 | 0.32 | 0.24 | 0.24 |
