# Supplementary material for: Anti-tobacco control industry strategies in Turkey
Source: BMC Public Health. 2018 Feb 26;18:282. doi: 10.1186/s12889-018-5071-z (PMC5828147; doi:10.1186/s12889-018-5071-z)
Supplement: Supplementary file 6 — Nominal sales revenues of cigarettes by price segment- sample (billion TL), 2005–2012. (DOCX 14 kb) [file 12889_2018_5071_MOESM6_ESM.docx]

Additional file 6: Nominal sales revenues of cigarettes by price segment- sample (billion TL), 2005-2012.

|  | **Premium** | **Mid-priced** | **Economy** |
| --- | --- | --- | --- |
| **2005** | 4.55 | 0.85 | 2.93 |
| **2006** | 5.24 | 1.01 | 3.64 |
| **2007** | 5.40 | 1.60 | 4.32 |
| **2008** | 4.51 | 1.86 | 3.71 |
| **2009** | 4.92 | 2.57 | 4.36 |
| **2010** | 5.48 | 3.42 | 3.96 |
| **2011** | 5.81 | 3.78 | 3.76 |
| **2012** | 7.04 | 4.81 | 3.97 |
