# Supplementary material for: Anti-tobacco control industry strategies in Turkey
Source: BMC Public Health. 2018 Feb 26;18:282. doi: 10.1186/s12889-018-5071-z (PMC5828147; doi:10.1186/s12889-018-5071-z)
Supplement: Supplementary file 7 — Retail sales volumes of cigarettes by price segment- sample (billion packs), 2005–2012. (DOCX 14 kb) [file 12889_2018_5071_MOESM7_ESM.docx]

Additional file 7: Retail sales volumes of cigarettes by price segment- sample (billion packs), 2005-2012.

|  | **Premium** | **Mid-priced** | **Economy** |
| --- | --- | --- | --- |
| **2005** | 1.19 | 0.31 | 1.60 |
| **2006** | 1.19 | 0.32 | 1.62 |
| **2007** | 1.14 | 0.46 | 1.76 |
| **2008** | 0.91 | 0.50 | 1.38 |
| **2009** | 0.91 | 0.63 | 1.38 |
| **2010** | 0.77 | 0.62 | 0.89 |
| **2011** | 0.79 | 0.66 | 0.81 |
| **2012** | 0.87 | 0.74 | 0.73 |
