# Supplementary material for: Anti-tobacco control industry strategies in Turkey
Source: BMC Public Health. 2018 Feb 26;18:282. doi: 10.1186/s12889-018-5071-z (PMC5828147; doi:10.1186/s12889-018-5071-z)
Supplement: Supplementary file 8 — Nominal weighted prices per pack of cigarettes by price segment (TL), 2005–2012. (DOCX 14 kb) [file 12889_2018_5071_MOESM8_ESM.docx]

Additional file 8: Nominal weighted prices per pack of cigarettes by price segment (TL), 2005-2012.

|  | **Premium** | **Mid-priced** | **Economy** |
| --- | --- | --- | --- |
| **2005** | 3.83 | 2.74 | 1.84 |
| **2006** | 4.41 | 3.20 | 2.25 |
| **2007** | 4.72 | 3.50 | 2.46 |
| **2008** | 4.94 | 3.75 | 2.69 |
| **2009** | 5.39 | 4.07 | 3.17 |
| **2010** | 7.08 | 5.48 | 4.44 |
| **2011** | 7.35 | 5.73 | 4.66 |
| **2012** | 8.12 | 6.50 | 5.41 |
