# Supplementary material for: Anti-tobacco control industry strategies in Turkey
Source: BMC Public Health. 2018 Feb 26;18:282. doi: 10.1186/s12889-018-5071-z (PMC5828147; doi:10.1186/s12889-018-5071-z)
Supplement: Supplementary file 9 — Real weighted prices per pack of cigarettes by price segment (TL), 2005–2012. (DOCX 14 kb) [file 12889_2018_5071_MOESM9_ESM.docx]

Additional file 9: Real weighted prices per pack of cigarettes by price segment (TL), 2005-2012.

|  | **Premium** | **Mid-priced** | **Economy** |
| --- | --- | --- | --- |
| **2005** | 3.12 | 2.24 | 1.50 |
| **2006** | 3.28 | 2.38 | 1.67 |
| **2007** | 3.24 | 2.40 | 1.69 |
| **2008** | 3.08 | 2.34 | 1.68 |
| **2009** | 3.15 | 2.38 | 1.85 |
| **2010** | 3.90 | 3.02 | 2.44 |
| **2011** | 3.66 | 2.85 | 2.32 |
| **2012** | 3.81 | 3.05 | 2.54 |
