# Supplementary material for: Anti-tobacco control industry strategies in Turkey
Source: BMC Public Health. 2018 Feb 26;18:282. doi: 10.1186/s12889-018-5071-z (PMC5828147; doi:10.1186/s12889-018-5071-z)
Supplement: Supplementary file 10 — Before-tax (net) nominal weighted prices per pack of cigarettes by price segment, (TL) 2005–2012. (DOCX 14 kb) [file 12889_2018_5071_MOESM10_ESM.docx]

Additional file 10: Before-tax (net) nominal weighted prices per pack of cigarettes by price segment, (TL) 2005-2012

|  | **Premium** | **Mid-priced** | **Economy** |
| --- | --- | --- | --- |
| **2005** | 1.02 | 0.73 | 0.49 |
| **2006** | 1.18 | 0.86 | 0.60 |
| **2007** | 1.26 | 0.94 | 0.66 |
| **2008** | 1.32 | 1.00 | 0.72 |
| **2009** | 1.44 | 1.09 | 0.85 |
| **2010** | 1.54 | 1.19 | 0.97 |
| **2011** | 1.45 | 1.13 | 0.92 |
| **2012** | 1.60 | 1.28 | 1.07 |
