# Supplementary material for: Anti-tobacco control industry strategies in Turkey
Source: BMC Public Health. 2018 Feb 26;18:282. doi: 10.1186/s12889-018-5071-z (PMC5828147; doi:10.1186/s12889-018-5071-z)
Supplement: Supplementary file 11 — Before-tax (net) real weighted prices per pack of cigarettes by price segment, (TL) 2005–2012. (DOCX 14 kb) [file 12889_2018_5071_MOESM11_ESM.docx]

Additional file 11: Before-tax (net) real weighted prices per pack of cigarettes by price segment, (TL) 2005-2012

|  | **Premium** | **Mid-priced** | **Economy** |
| --- | --- | --- | --- |
| **2005** | 0.83 | 0.60 | 0.40 |
| **2006** | 0.88 | 0.64 | 0.45 |
| **2007** | 0.87 | 0.64 | 0.45 |
| **2008** | 0.82 | 0.63 | 0.45 |
| **2009** | 0.84 | 0.64 | 0.50 |
| **2010** | 0.85 | 0.66 | 0.53 |
| **2011** | 0.72 | 0.56 | 0.46 |
| **2012** | 0.75 | 0.60 | 0.50 |
